# Anxiety and Depression in People with Eczema or Psoriasis: A Comparison of Associations in UK Biobank and Linked Primary Care Data

**DOI:** 10.2147/CLEP.S417176

**Published:** 2023-08-07

**Authors:** Julian Matthewman, Kathryn E Mansfield, Joseph F Hayes, Elizabeth I Adesanya, Catherine H Smith, Amanda Roberts, Sinéad M Langan, Alasdair D Henderson

**Affiliations:** 1 Department of Non-Communicable Disease Epidemiology, London School of Hygiene and Tropical Medicine, London, UK; 2 Division of Psychiatry, University College London, London, UK; 3 St John’s Institute of Dermatology, Kings College London, London, UK

**Keywords:** eczema, psoriasis, anxiety, depression, ascertainment, cross-sectional study, data linkage, UK Biobank, electronic health records

## Abstract

**Introduction:**

Previous research has shown associations between eczema and psoriasis and anxiety and depression. We investigated whether associations are consistent across different settings of ascertainment for depression and anxiety, including interview and survey responses from UK Biobank (a large longitudinal cohort recruiting individuals aged 40–69 years between 2006–2010), and linked primary care data, with the aim of drawing more reliable conclusions through triangulation.

**Methods:**

In cross-sectional studies, we estimated associations between eczema or psoriasis and anxiety or depression, defining anxiety or depression as 1) self-reported previous diagnosis at UK Biobank recruitment interview; 2) PHQ-9/GAD-7 score indicating depression or anxiety from a UK Biobank mental health follow-up survey in 2016; and 3) diagnosis in linked primary care electronic health record data.

**Results:**

We analysed 230,047 people with linked Biobank and primary care data. We found poor agreement between the data sources for eczema, psoriasis, anxiety, and depression. Eg, 9474 had a previous eczema diagnosis in primary care data, 4069 self-reported previous eczema diagnosis at the UK biobank interview, and 1536 had eczema in both data sources (for depression 40,455; 13,320; and 9588 respectively). Having eczema or psoriasis (recorded in primary care or baseline interview) was associated with higher odds of anxiety and depression. Eg, the adjusted odds ratio for depression comparing those with eczema to those without was greater than 1 when defining the outcome from 1) the recruitment interview (1.36, 95% confidence interval 1.27–1.45); 2) the follow-up survey (1.24, 1.09–1.39), and 3) primary care records (1.56, 1.50–1.62).

**Discussion:**

Our findings support increased prevalence of mental illness in people with psoriasis and eczema across multiple data sources, which should be considered in planning of mental health services. However, we found poor agreement in disease ascertainment between settings, with implications for data interpretation in electronic health records.

## Introduction

Atopic eczema (referred to as eczema throughout) is common, affecting up to 10% of adults, while psoriasis affects 1–2% of adults in the UK.^1^,^2^ Previous evidence, including from cohort studies using UK primary care electronic health records, has found that existing eczema and psoriasis are associated with newly reported anxiety and depression.^3–6^

To increase trust in associations found between eczema/psoriasis and anxiety/depression it is important to triangulate findings using different approaches.^7^ Firstly, the effects should be demonstrated across multiple types of data sources, eg, both routinely collected health records and survey data. Secondly, for diseases that are heterogenous in their severity, progression, and real-world diagnosis context, it is important to demonstrate similar effects using multiple disease definitions (eg, clinician diagnosis, self-report). Differences in the extent to which conditions are captured in different data sources may be explained by social desirability, recall bias, consultation behaviour, or differences in clinicians’ coding behaviour (eg, due to changes in how general practitioners record mental illness).^8^

Defining mental health outcomes, in UK Biobank and elsewhere, is complex, making it important that multiple measures for ascertaining mental illness status are used.^9^ Considering different mental illness outcome definitions is also especially important in the context of studying associations with skin disease exposures. For example, while it is likely that anxiety and depression are underreported and underdiagnosed in primary care in the general population,^10^ it is possible that any underreporting of anxiety/depression is worse in people with eczema/psoriasis; consultations may focus on skin conditions, as there is evidence that those presenting with physical symptoms (eg, symptoms of skin conditions) are less likely to have their mental illness detected or prioritised.^11–14^

UK Biobank is a large UK longitudinal cohort study established in 2006 that is regularly used for observational research of skin diseases,^15^ and mental illnesses.^16^,^17^ UK Biobank recently linked a proportion of their cohort to primary care data, affording the opportunity to look at associations (eg, between a chronic conditions like eczema/psoriasis, and adverse health outcomes like anxiety/depression) in and beyond primary care, within the same population.^18^ We used Biobank baseline data, follow-up mental health questionnaire data from 2016, and linked primary care electronic health record data, all from the same study population, with the aim of estimating the associations between eczema/psoriasis and anxiety/depression across multiple settings of disease ascertainment to increase confidence in the previously observed association.

## Methods

### Study Population

We used data from UK Biobank, a database including approximately half a million participants aged 40–69 years at recruitment between 2006 and 2010. Of these, we included only participants with linked primary care data (n=230,047).

### Exposure and Outcome Measurement

We defined eczema and psoriasis exposure using both UK Biobank recruitment interview responses (self-reported previous diagnosis of serious illnesses or disabilities; as was also done in previous UK Biobank studies),^19^,^20^ and primary care records based on a previously validated algorithm (one eczema diagnostic code and two records for eczema therapy recorded on separate days; one diagnostic code for psoriasis).^21^

We defined anxiety and depression outcomes in three ways: 1) UK Biobank recruitment interview responses (self-reported previous diagnosis of serious illnesses or disabilities) coded as depression or anxiety/panic attacks (Appendix Section “Exposure & Outcomes in UK Biobank”, Supplementary Table 1); 2) Biobank 2016 mental health follow-up survey response derived PHQ-9 (Patient Health Questionnaire)^22^ and GAD-7 (Generalised Anxiety Disorder Assessment)^23^ scores for depression and anxiety in the two weeks before the 2016 mental health follow-up survey, with scores of 10 or more considered as being indicative of present anxiety/depression (Appendix Section “PHQ-9/GAD-7 scores”); 3) primary care morbidity coding defined based on a single morbidity code for anxiety or depression, including diagnoses and symptoms of anxiety/depression, recorded prior to the Biobank interview/2016 mental health follow-up survey (primary care data available from approximately 1990 onwards).

To calculate PHQ-9/GAD-7 scores, the respondent is asked to judge “Over the last 2 weeks, how often have you been bothered by any of the following problems?” with nine/seven responses taken for PHQ-9/GAD-7 (eg, “Little interest or pleasure in doing things” for PHQ-9; “Becoming easily annoyed or irritable” for GAD-7). The overall scores are calculated by assigning scores of 0 (“not at all”), 1 (“several days”), 2 (“more than half the days”), and 3 (“nearly every day”), and adding together the scores for the nine/seven questions.

We used lists of primary care morbidity codes for diagnoses, symptoms, and prescriptions to identify eczema/psoriasis and anxiety/depression in primary care data. We used morbidity code lists used in previous electronic health record research developed with input from UK-practicing clinicians (for more detail see Appendix Section “Codelists”, Supplementary Table 2).^4^,^21^,^24–26^ All data management and statistical analysis code is available on GitHub (repository to be published together with manuscript).

### Statistical Analysis

We described the baseline characteristics of our study population (Ie, the subset of the Biobank cohort with linked primary care data), and of the entire Biobank cohort by linkage status. We described the number of people with and without eczema/psoriasis who had anxiety/depression separately for all exposure and outcome pairs (ie, different exposure/outcome definitions). We additionally described how many people self-reported anxiety/depression symptoms that occurred any time before the Biobank mental health follow-up survey. We assessed agreement between recruitment interview and primary care data for all exposures (eczema/psoriasis) and outcomes (anxiety/depression).

**Figure 1:**
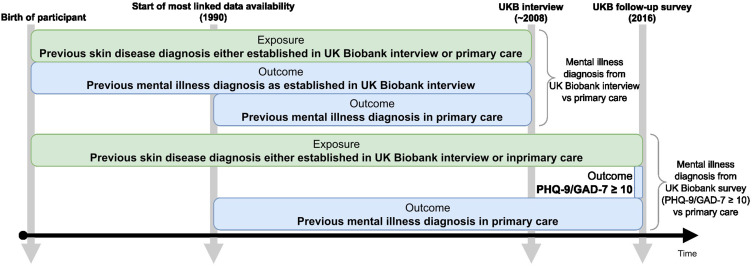
Timeline showing when exposure and outcome for both cross-sectional comparisons were defined and the timeframes from when the actual diagnoses/self-reports would be from. Most participants with primary care data only had data available from 1990 onwards, whereas self-reported previous diagnoses could potentially have occurred before that time. In green: The exposure (eczema/psoriasis) was defined as a previous doctor’s diagnosis either reported at the UK Biobank interview around 2008 or at least 1 code for eczema diagnosis and 2 codes for eczema treatments on different days in primary care data. Only data from before the UK Biobank interview or the UK Biobank follow-up survey was used. In blue: The outcome (anxiety/depression) was defined as a previous doctor’s diagnosis reported at the UK Biobank interview around 2008, at least 1 diagnosis code in primary care data, or a PHQ-9/GAD-7 score of more than 10 at the UK Biobank mental health follow-up survey.

We conducted cross-sectional studies, using logistic regression to estimate the association (odds ratios [OR] and 95% confidence intervals [95% CI]) between eczema/psoriasis and anxiety/depression. We adjusted models for key potential confounders (age, sex, deprivation, ethnicity) (Appendix Section “Covariates”). We estimated odds ratios comparing the odds of each anxiety/depression outcome definition (self-reported diagnosis at initial interview; PHQ-9/GAD-7 ≥10 in mental health follow-up survey; coded in primary care data) in people with eczema/psoriasis (captured in either electronic health records or on baseline Biobank survey) compared to people without eczema/psoriasis (Figure 1). All code is available online.^27^

## Results

**Figure 2:**
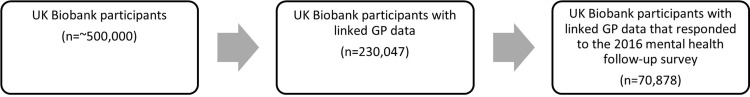
Participant flow.

We included 230,047 people from Biobank with linked primary care data (Figure 2). The study population was aged 40–69 years at recruitment, included more women than men (55% female), and was mostly of people who reported their ethnicity to be “British” (89%). Our study population (people with primary care data linkage) had similar distribution of baseline characteristics to those without linkage; those within our study population that responded to the mental health survey where from more affluent areas and were less likely to be retired than those who did not respond to the survey (Supplementary Figure 1, Supplementary Table 3).

### Agreement Between Data Sources

**Figure 3:**
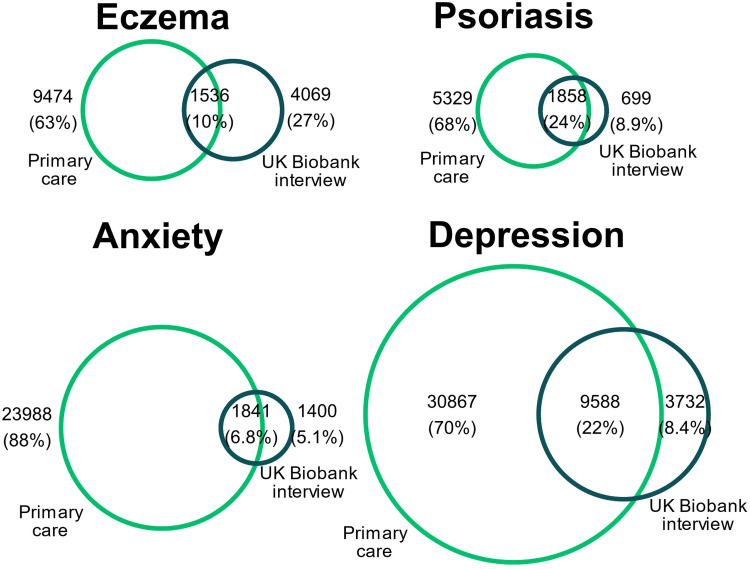
Exposure and outcome definition agreement between UK Biobank interview at recruitment, and primary care records. For each exposure/outcome the Venn diagram show the number of people that identified the condition in their baseline interview and the number of people who have a corresponding record in primary care.

More individuals were identified as having previous eczema, psoriasis, depression, and anxiety in their primary care records than was reported on recruitment interview (eg, 11,010 had an eczema record in their electronic health records, compared to 5605 reporting previous eczema on recruitment interview; 7187 vs 2557 for psoriasis; 40,455 vs 13,326 for depression; 49,268 vs 3242 for anxiety) (Table 1). ‬A minority of participants met the disease definition in both data sources: eczema 8%, psoriasis 25%, depression 22%, anxiety 7% (Figure 3).

**Table 1:** UK Biobank Recruitment Interview Compared to Primary Care Morbidity Coding at or Before Recruitment

	Anxiety as Defined in		Depression as Defined in	
	Interview^a^	Primary Care^b^	Interview^a^	Primary Care^b^
**No Eczema** n=214,968 (100%)	2971 (1.4%)	23,439 (11%)	12,189 (5.7%)	36,783 (17%)
**Eczema** n=15,079 (100%)	271 (1.8%)	2390 (16%)	1137 (7.5%)	3672 (24%)
**No Psoriasis** n=222,139 (100%)	3101 (1.4%)	24,677 (11%)	12,825 (5.77%)	38,691 (17%)
**Psoriasis** n=7886 (100%)	141 (1.8%)	1152 (15%)	500 (6.34%)	1758 (22%)

**Notes**: Number of people with and without eczema/psoriasis (based on recruitment interview and/or primary care data up to recruitment) who have anxiety/depression (and percentage of people with anxiety/depression of total people with/without eczema/psoriasis) as defined in ^a^Reponses from UKB interview (around 2008; people were asked if they had ever been diagnosed by a doctor with any serious illnesses), ^b^Primary care data up to date of UKB interview. Percentages are row percentages.

A total of 70,878 of those with primary care records responded to the 2016 mental health follow-up survey, of whom 4113 (5.8%) had a PHQ-9 score indicating current depression, and 3177 (4.4%) had a GAD-7 score indicating current anxiety; 10,999 (15.5%)‬ ever‬ had a primary care record indicating depression, and 7008 (9.9%) a recording indicating anxiety up to the date of the mental health follow-up survey (Table 2).

**Table 2:** UK Biobank Mental Health Follow-Up Survey (in 2016) Compared to Primary Care Morbidity Coding at or Before Survey

	Anxiety as Defined in		Depression as Defined in	
	Survey (GAD-7 ≥ 10)^a^	Primary Care^b^	Survey (PHQ-9 ≥ 10)^a^	Primary Care^b^
**No Eczema** n=66,253 (100%)	2937 (4.5%)	6371 (9.6%)	3800 (5.8%)	10,005 (15%)
**Eczema** n=4628 (100%)	240 (5.3%)	637 (14%)	313 (6.9%)	994 (21%)
**No Psoriasis** n=68,584 (100%)	3060 (4.5%)	6715 (9.8%)	3962 (5.9%)	10,553 (15%)
**Psoriasis** n=2294 (100%)	117 (5.2%)	293 (13%)	151 (6.7%)	446 (19%)

**Notes**: Number of people with and without eczema/psoriasis (based on recruitment interview and/or primary care data up to survey) who have anxiety/depression (and percentage of people with anxiety/depression of total people with/without eczema/psoriasis) as defined in ^a^UKB follow-up survey, as a score of ≥10 in the PHQ-9 score for depression/the GAD-7 score for anxiety, which take into account symptoms in the 2 weeks leading up to the 2016 UKB follow-up survey, ^b^Linked GP data up to date of UKB survey, including only people who answered the survey. Percentages are row percentages.

### Eczema/Psoriasis and Anxiety/Depression

Having eczema or psoriasis was associated with higher odds of having both anxiety and depression, regardless of the method used to define the mental illness (Biobank interview, mental health survey, or primary care data). The adjusted (age, sex, deprivation, ethnicity) odds ratios for the association between eczema or psoriasis and anxiety or depression were larger when defining anxiety or depression using primary care records compared to UK Biobank interview/survey data. This was true both for the comparison at recruitment (eg exposure: eczema, outcome: depression defined in Biobank interview, OR 1.36, 95% CI 1.27–1.45; outcome defined in prior primary care data, OR 1.56, 95% CI 1.50–1.62) and the comparison at the mental health survey (eg, exposure: eczema, outcome: depression defined from Biobank mental health survey: OR 1.24, 95% CI 1.09–1.39; outcome defined in pre-survey primary care records: OR 1.50, 95% CI 1.39–1.63) (Table 3).

**Table 3:** Odds Ratios from Logistic Regression by Data Source of Outcome Definition

	Outcome Definition^b^	Exposure: Eczema^a^		Exposure: Psoriasis^a^	
		OR (95% CI)^c^	n^d^	OR (95% CI)^c^	n^d^
**UK Biobank**					
Depression	Interview-reported diagnosis	1.36 (1.27–1.45)	229,393	1.11 (1.01–1.21)	229,371
Anxiety	Interview-reported diagnosis	1.30 (1.14–1.47)	229,393	1.29 (1.08–1.52)	229,371
Depression	PHQ-9 ≥ 10 (survey)	1.24 (1.09–1.39)	69,420	1.19 (1.00–1.41)	69,417
Anxiety	GAD-7 ≥ 10 (survey)	1.20 (1.05–1.37)	69,737	1.19 (0.98–1.44)	69,734
**Linked GP data**					
Depression	≥ 1 diagnosis pre-interview	1.56 (1.50–1.62)	229,378	1.38 (1.31–1.46)	229,356
Anxiety	≥ 1 diagnosis pre-interview	1.53 (1.46–1.60)	229,390	1.38 (1.29–1.47)	229,368
Depression	≥ 1 diagnosis pre-survey	1.50 (1.39–1.63)	64,499	1.37 (1.22–1.54)	64,496
Anxiety	≥ 1 diagnosis pre-survey	1.43 (1.30–1.58)	64,502	1.39 (1.21–1.59)	64,499

**Notes**: ^a^Exposures defined using self-reported previous diagnosis at the UK Biobank recruitment interview, or through records in linked GP data prior to the timepoint (at least 1 diagnosis + 2 prescription codes on separate days for eczema; 1 diagnosis for psoriasis). ^b^At the Initial interview timepoint (in grey), outcomes are defined either as a self-reported previous doctor’s diagnosis, or at least 1 diagnosis code in linked GP data prior to the interview. At the 2016 mental health follow-up survey (70,878 responded), outcomes are defined either as a score of ≥10 in the PHQ-9 score for depression/the GAD-7 score for anxiety, which take into account symptoms in the 2 weeks prior to the survey, or at least 1 diagnosis code in linked GP data prior to the follow-up survey. ^c^Odds ratios (95% confidence intervals) estimated from logistic regression for having a mental illness (adjusted for age, sex, deprivation and ethnicity) comparing people with the respective skin disease to people without the respective skin disease. ^d^Number of observations that went into the model. Observations with missing values were dropped. “Prefer not to answer” and “Do not know” were treated as missing values. For the follow-up survey timepoint, only used GP data where all of the questions of the mental health follow-up survey were answered.

## Discussion

We found poor agreement between populations of people with eczema, psoriasis, anxiety or depression as captured in UK Biobank versus linked primary care data. This lack of agreement in diagnoses between primary care and survey data demonstrates that, depending on the specific disease, it is likely that there will be differential capture of conditions depending on data sources and setting of ascertainment. Despite low agreement, we found consistent evidence from primary care and UK Biobank data that people with two common inflammatory skin conditions – eczema and psoriasis – are more likely to experience anxiety and depression regardless of whether we captured anxiety/depression in primary care records or through UK Biobank interview/survey data (albeit with weaker strengths of associations with interview/survey data). This is consistent with previous findings from other studies, including those in UK primary care data.^3^,^4^

We found a lower prevalence of all exposures (eczema/psoriasis) and outcomes (anxiety/depression) in UK Biobank survey/interview data compared to linked primary care records. The interview question at baseline in UK Biobank was “[…] you have been told by a doctor that you have other serious illnesses or disabilities, could you now tell me what they are?”. Many people with a record in primary care of one of eczema, psoriasis, anxiety or depression, did not report this at the interview (eg of 9474 people with a primary care record for eczema, 1536 also reported this at the interview), which may suggest that only the most severe cases of eczema/psoriasis and anxiety/depression were reported in UK Biobank. Additionally, individuals may not report their mental illness in an interview due to social desirability bias.^28^ For mental health outcome measures, poor agreement between UK Biobank and linked data sources has been previously described,^9^ and for psoriasis, previous research has recommended using UK Biobank in conjunction with another data source to improve accuracy.^29^

### Strengths and Weaknesses

The major strength of this study is that we have applied consistent study design and analyses to the same population with information from three different sources (UK Biobank interview, UK Biobank survey and primary care) and have found consistent associations between eczema or psoriasis and anxiety or depression.

Given the cross-sectional design of our study, we were not able to consider whether eczema or psoriasis preceded anxiety or depression, therefore we were unable to assess temporality. In addition, while we adjusted for key confounders of associations between skin conditions and mental illness (age, sex, deprivation, ethnicity), it is likely unmeasured confounding remains, especially with regard to comorbidities. However, we selected a more parsimonious model for two reasons: 1) we were primarily interested in the comparability of estimates between data sources and not causal inference, so a simpler model specification was preferred; and 2) we wanted to reduce the influence of covariate misclassification between data sources. We found that the agreement between all four exposure/outcome definitions was low, so it is likely that this problem would exist for covariates as well. We therefore did not include other covariates in our analysis to limit possible explanations for differences in our findings between the data sources.

Another limitation of our findings, especially in comparison to research from UK wide primary care records,^4^ is that the UK Biobank population is subject to strong selection pressures.^30^ In general, the UK Biobank cohort are from a certain age range (40–69 at recruitment in 2006–2010), and predominantly of white ethnicity. The select Biobank population limits the generalisability of our findings to the wider UK population. However, selection bias will not limit internal validity, as we were comparing results from Biobank interviews and surveys to the linked primary care records for the same individuals. Despite the highly selected population, research using UK Biobank data has been previously found to produce generalisable estimates of risk factor associations.^31^

Selection bias is a particular limitation of the analysis of the 2016 mental health questionnaire (31% of the study population). We believe, however, that the selection would likely be non-differential by eczema/psoriasis status, supported by the similar distribution of eczema (2.7% vs 2.3%) and psoriasis (1.1% vs 1.1%) we saw at recruitment in those who did and did not respond to the survey. However, the results from the mental health questionnaire data may be inconsistent with findings from the whole UK Biobank or UK population. Despite the select population, even in this restricted sample measuring recent anxiety or depression we found worse scores in people with eczema and psoriasis.

While our findings were consistent across different mental illness definitions, including using the PHQ-9/GAD-7 scores, we acknowledge that PHQ-9/GAD-7 instruments will only capture recent anxiety and depression symptoms and may not be directly comparable to having a previous anxiety/depression diagnosis, which the other definitions were capturing. While self-reported symptoms that were used to derive PHQ-9 and GAD-7 scores will not be subject to the same kind of differences in ascertainment that can occur in routinely collected health data, they do only capture current disease.

We found stronger associations (greater magnitude odds ratios) in our results from primary care data only, compared to those from UK Biobank interview/survey data. However, both primary care-based and interview/survey-based estimates may be subject to different biases that may explain the higher magnitude ORs in primary care data in ways that are unrelated to the association between eczema or psoriasis and anxiety or depression. Results from primary care data may be subject to differential ascertainment of anxiety or depression between people with and without skin conditions. People with eczema or psoriasis may consult their GP more frequently, giving more opportunity to have other conditions diagnosed. Alternatively, results from Biobank recruitment interview and follow up mental health survey may be influenced by differential capture of anxiety or depression outcomes between those with and without eczema or psoriasis. It is possible that there will be differences in how people with and without eczema or psoriasis answered interview questions and self-defined their symptoms. It is not possible to differentiate these mechanisms in this study; however, our work does demonstrate that the choice of data source and method of outcome assessment will influence observed associations.

### Implications and Future Research

Our research question was “Are eczema and psoriasis associated with depression or anxiety?”. We found an association in the same population across multiple settings of disease ascertainment, which increases confidence in the existence of this association as the question was addressed using a number of different approaches.^7^ Taken together with findings from the existing body of literature on this topic,^3^ this motivates improved planning of mental health services for people with eczema and psoriasis.

Our findings suggest that the method of ascertainment of study conditions influences what is captured in observational epidemiological studies regardless of whether these use electronic health records or survey/interview data. These key differences in study definitions may impact interpretability when comparing findings from UK Biobank interview/survey data alone to those where diseases are defined in primary care records. In particular, from UK Biobank data it may only be possible to capture serious or currently active eczema/psoriasis and anxiety/depression. We therefore recommend future research to better understand the phenotypic differences between groups with the same health condition identified from different health care record data sources.

We found that associations between eczema or psoriasis and anxiety or depression were of a slightly lower magnitude when using interview/survey responses to define anxiety or depression compared to using morbidity coded primary care records. Further research into who does and does not consult their GP with these symptoms is necessary to target interventions and help effectively.

## Conclusion

We found that capturing the same health conditions (eczema/psoriasis/anxiety/depression) in primary care records and interview/survey data in the same group of individuals had poor agreement. Despite these differences in who was identified as having eczema/psoriasis and anxiety/depression in our study, we consistently found evidence of an association between eczema/psoriasis and anxiety/depression, regardless of how anxiety/depression were defined, including as self-reported previous doctors’ diagnosis, current adverse mental health as captured by PHQ-9/GAD-7 questionnaires, or previous records in primary care data.

## References

[1] Barbarot S, Auziere S, Gadkari A, et al. Epidemiology of atopic dermatitis in adults: results from an international survey. *Allergy*. 2018;73(6):1284–1293. doi:10.1111/all.13401 29319189

[2] Parisi R, Symmons DPM, Griffiths CEM, Ashcroft DM. Global epidemiology of psoriasis: a systematic review of incidence and prevalence. *J Investig Dermatol*. 2013;133(2):377–385. 23014338 10.1038/jid.2012.339

[3] Bao Q, Chen L, Lu Z, et al. Association between eczema and risk of depression: a systematic review and meta-analysis of 188,495 participants. *J Affect Disord*. 2018;238:458–464. 29929155 10.1016/j.jad.2018.05.007

[4] Schonmann Y, Mansfield KE, Hayes JF, et al. Atopic eczema in adulthood and risk of depression and anxiety: a population-based cohort study. *J Allergy Clin Immunol*. 2020;8(1):248–257.e16. 10.1016/j.jaip.2019.08.030 PMC6947493 31479767

[5] Kurd SK, Troxel AB, Crits-Christoph P, Gelfand JM. The risk of depression, anxiety and suicidality in patients with psoriasis: a population-based cohort study. *Arch Dermatol*. 2010;146(8):891–895. 20713823 10.1001/archdermatol.2010.186 PMC2928071

[6] Ferreira BR, Pio-Abreu JL, Reis JP, Figueiredo A. Analysis of the prevalence of mental disorders in psoriasis: the relevance of psychiatric assessment in dermatology. *Psychiatr Danub*. 2017;29(4):401–406. 29197196 10.24869/psyd.2017.401

[7] Lawlor DA, Tilling K, Davey Smith G. Triangulation in aetiological epidemiology. *Int J Epidemiol*. 2016;45(6):1866–1886. 28108528 10.1093/ije/dyw314 PMC5841843

[8] Mitchell C, Dwyer R, Hagan T, Mathers N. Impact of the QOF and the NICE guideline in the diagnosis and management of depression: a qualitative study. *Br J Gen Pract*. 2011;61(586):e279–89. 21619752 10.3399/bjgp11X572472 PMC3080233

[9] Davis KAS, Cullen B, Adams M, et al. Indicators of mental disorders in UK Biobank—a comparison of approaches. *Int J Methods Psychiatr Res*. 2019;28(3):e1796. 31397039 10.1002/mpr.1796 PMC6877131

[10] Bell RA, Franks P, Duberstein PR, et al. Suffering in silence: reasons for not disclosing depression in primary care. *Ann Fam Med*. 2011;9(5):439–446. doi:10.1370/afm.1277 21911763 PMC3185469

[11] Menchetti M, Belvederi Murri M, Bertakis K, Bortolotti B, Berardi D. Recognition and treatment of depression in primary care: effect of patients’ presentation and frequency of consultation. *J Psychosom Res*. 2009;66(4):335–341. 19302892 10.1016/j.jpsychores.2008.10.008

[12] Pfaff JJ, Almeida OP. A cross-sectional analysis of factors that influence the detection of depression in older primary care patients. *Aust N Z J Psychiatry*. 2005;39(4):262–265. 15777363 10.1080/j.1440-1614.2005.01563.x

[13] Nuyen J, Volkers AC, Verhaak PFM, Schellevis FG, Groenewegen PP, den Bos GAM V. Accuracy of diagnosing depression in primary care: the impact of chronic somatic and psychiatric co-morbidity. *Psychol Med*. 2005;35(8):1185–1195. 16116944 10.1017/s0033291705004812

[14] Aragonès E, Piñol JL, Labad A, Folch S, Mèlich N. Detection and management of depressive disorders in primary care in Spain. *Int J Psychiatry Med*. 2004;34(4):331–343. 15825583 10.2190/N835-FDYX-2E2E-V8XM

[15] Budu-Aggrey A, Brumpton B, Tyrrell J, et al. Evidence of a causal relationship between body mass index and psoriasis: a Mendelian randomization study. *PLoS Med*. 2019;16(1):e1002739. 30703100 10.1371/journal.pmed.1002739 PMC6354959

[16] Kandola AA, Del Pozo Cruz B, Osborn DPJ, Stubbs B, Choi KW, Hayes JF. Impact of replacing sedentary behaviour with other movement behaviours on depression and anxiety symptoms: a prospective cohort study in the UK Biobank. *BMC Med*. 2021;19(1):133. 34134689 10.1186/s12916-021-02007-3 PMC8210357

[17] Mutz J, Hoppen TH, Fabbri C, Lewis CM. Anxiety disorders and age-related changes in physiology. *Br J Psychiatr*. 2022;221(3):528–537. 10.1192/bjp.2021.189 PMC7613411 35048844

[18] Wilkinson T, Schnier C, Bush K, et al.; On behalf of Dementias Platform UK and UK Biobank. Identifying dementia outcomes in UK Biobank: a validation study of primary care, hospital admissions and mortality data. *Eur J Epidemiol*. 2019;34(6):557–565. 30806901 10.1007/s10654-019-00499-1 PMC6497624

[19] Johansson Å, Rask-Andersen M, Karlsson T, Ek WE. Genome-wide association analysis of 350 000 Caucasians from the UK Biobank identifies novel loci for asthma, hay fever and eczema. *Hum Mol Genet*. 2019;28(23):4022–4041. 31361310 10.1093/hmg/ddz175 PMC6969355

[20] Ek WE, Karlsson T, Hernándes CA, Rask-Andersen M, Johansson Å. Breast-feeding and risk of asthma, hay fever, and eczema. *J Allergy Clin Immunol*. 2018;141(3):1157–1159.e9. 29132959 10.1016/j.jaci.2017.10.022

[21] Abuabara K, Magyari AM, Hoffstad O, et al. Development and validation of an algorithm to accurately identify atopic eczema patients in primary care electronic health records from the UK. *J Investig Dermatol*. 2017;137(8):1655–1662. 28428130 10.1016/j.jid.2017.03.029 PMC5883318

[22] Kroenke K, Spitzer RL, Williams JB. The PHQ-9: validity of a brief depression severity measure. *J Gen Intern Med*. 2001;16(9):606–613. 11556941 10.1046/j.1525-1497.2001.016009606.x PMC1495268

[23] Spitzer RL, Kroenke K, Williams JBW, Löwe B. A brief measure for assessing generalized anxiety disorder: the GAD-7. *Arch Intern Med*. 2006;166(10):1092–1097. 16717171 10.1001/archinte.166.10.1092

[24] Silverwood RJ, Forbes HJ, Abuabara K, et al. Severe and predominantly active atopic eczema in adulthood and long term risk of cardiovascular disease: population based cohort study. *BMJ*. 2018;2018:k1786. 10.1136/bmj.k1786 PMC6190010 29792314

[25] Lowe KE, Mansfield KE, Delmestri A, et al. Atopic eczema and fracture risk in adults: a population-based cohort study. *J Allergy Clin Immunol*. 2020;145(2):563–571.e8. 31757515 10.1016/j.jaci.2019.09.015 PMC7014587

[26] Matthewman J, Mansfield KE, Prieto-Alhambra D, et al. Atopic eczema–associated fracture risk and oral corticosteroids: a population-based cohort study. *J Allergy Clin Immunol*. 2021;2021:154. 10.1016/j.jaip.2021.09.026 PMC7612204 34571200

[27] Henderson A, Matthewman J. hendersonad/2022_biobank: first release for paper submission. Zenodo; 2023. Available from: https://zenodo.org/record/7767171. Accessed July 25, 2027.

[28] Latkin CA, Edwards C, Davey-Rothwell MA, Tobin KE. The relationship between social desirability bias and self-reports of health, substance use, and social network factors among urban substance users in Baltimore, Maryland. *Addict Behav*. 2017;73:133–136. 28511097 10.1016/j.addbeh.2017.05.005 PMC5519338

[29] Saklatvala JR, Hanscombe KB, Mahil SK, et al. Genetic validation of psoriasis phenotyping in UK Biobank supports the utility of self-reported data and composite definitions for large genetic and epidemiological studies. *J Invest Dermatol*. 2023;20:254.10.1016/j.jid.2023.02.01036870556

[30] Swanson JM. The UK Biobank and selection bias. *Lancet*. 2012;380(9837):110. 10.1016/S0140-6736(12)61179-9 22794246

[31] Batty GD, Gale CR, Kivimäki M, Deary IJ, Bell S. Comparison of risk factor associations in UK Biobank against representative, general population based studies with conventional response rates: prospective cohort study and individual participant meta-analysis. *BMJ*. 2020;368:m131. 32051121 10.1136/bmj.m131 PMC7190071

